# Infrequent Breakfast Consumption Is Associated with Higher Body Adiposity and Abdominal Obesity in Malaysian School-Aged Adolescents

**DOI:** 10.1371/journal.pone.0059297

**Published:** 2013-03-08

**Authors:** Abdullah Nurul-Fadhilah, Pey Sze Teo, Inge Huybrechts, Leng Huat Foo

**Affiliations:** 1 Program of Nutrition, School of Health Sciences, Universiti Sains Malaysia, Health Campus, Kubang Kerian, Kelantan, Malaysia; 2 Dietary Exposure Assessment Group, International Agency for Research on Cancer (IARC), Lyon, France; 3 Department of Public Health, Ghent University, Ghent, Belgium; The University of Texas M. D. Anderson Cancer Center, United States of America

## Abstract

Unhealthy dietary pattern increases the risk of obesity and metabolic disorders in growing children and adolescents. However, the way the habitual pattern of breakfast consumption influences body composition and risk of obesity in adolescents is not well defined. Thus, the aim of the present study was to assess any associations between breakfast consumption practices and body composition profiles in 236 apparently healthy adolescents aged 12 to 19 years. A self-administered questionnaire on dietary behaviour and lifestyle practices and a dietary food frequency questionnaire were used. Body composition and adiposity indices were determined using standard anthropometric measurement protocols and dual energy χ-ray absorptiometry (DXA). Mean age of the participants was 15.3±1.9 years. The majority of participants (71.2%) fell in the normal body mass index (BMI) ranges. Breakfast consumption patterns showed that only half of the participants (50%) were consuming breakfast daily. Gender-specific multivariate analyses (ANCOVA) showed that in both boys and girls, those eating breakfast at least 5 times a week had significantly lower body weight, body mass index (BMI), BMI z-scores, waist circumference, body fat mass and percent body fat (%BF) compared to infrequent breakfast eaters, after adjustment for age, household income, pubertal status, eating-out and snacking practices, daily energy intakes, and daily physical activity levels. The present findings indicate that infrequent breakfast consumption is associated with higher body adiposity and abdominal obesity. Therefore, daily breakfast consumption with healthy food choices should be encouraged in growing children and adolescents to prevent adiposity during these critical years of growth.

## Introduction

An increasing worldwide prevalence of childhood obesity is a major public health challenge. Excessive weight gain and obesity during childhood and adolescence not only increase the risk of a range of health problems in youth, but also of developing chronic diseases in later life [1], [2]. Although it has been shown that genetic factors play a major role in the risk of developing obesity, modifiable environmental factors such as dietary and lifestyle practices are also important in the increasing rates of childhood obesity [2]. Furthermore, many studies have identified the determinants and correlates of excessive weight gain and obesity during childhood and adolescence. Unhealthy diets such as high intakes of energy-dense foods, along with low levels of physical activity have been shown to significantly increase the risk of weight gain and obesity in children and adolescents [3], [4], [5]. However, there is little information about the relationship between specific dietary behaviors such as habitual breakfast consumption practices and adiposity among children and adolescents.

There is evidence suggesting that daily consumption of breakfast is associated with better foods choice, such as more fruit and vegetables, with consequently also better intakes of essential nutrients [6], [7]. However, studies on the influence of infrequent breakfast consumption on obesity or other health problems in growing children and adolescents have been inconclusive. Some have found that children who frequently skip breakfast have a higher risk of being obese compared to those who regularly consumed breakfast [8], [9], [10]. For example, Harding and colleagues found an association between breakfast skipping and obesity among adolescents in the United Kingdom [8]. On the other hand, studies in children and adolescents from Australia, Portugal and Saudi Arabia have reported no such positive association between infrequent breakfast consumption, and body composition and obesity risk [11], [12], [13]. Such inconsistency between studies may possibly be explained by differences in the definition used for breakfast skipping and also in the choice of dietary and lifestyle factors that were used as confounding variables.

Although it is generally agreed that breakfast is the most important meal of the day [14], there has been a gradual increase in the proportion of children and adolescents who reported that they regularly skipped breakfast [15]. A decline in regular breakfast consumption by children has also been reported in Asia [16], [17]. For instance, approximately 10% of school-aged children and adolescents in Hong Kong were reported to be skipping breakfast at least 4 times a week [16]. In Malaysia, it has been found that breakfast is the most frequently missed meal [17]. Because of this trend towards breakfast skipping in children and adolescents, and because of the possible health effects, it is important to determine whether this dietary pattern might affect body adiposity and abdominal obesity of Malaysian children and adolescents.

There is little published information on any relationship between infrequent breakfast consumption and body composition profiles in growing children in Asian countries. Therefore, the aim of the present study was to determine the influence of breakfast consumption patterns on body composition measurements among apparently healthy Malaysian school-aged male and female adolescents.

## Participants and Methods

### Ethics statement

This study was approved by the Research Human Ethics Committees of the Universiti Sains Malaysia (USM) for human studies. In addition, written informed consent was also obtained prior to the study from both the participants and their parents or guardians.

### Study design

The present population-based study was carried-out in Kota Bharu, Kelantan, which is located in the Northeastern region of the Peninsular Malaysia. A convenience sample of 237 school-aged Malay adolescents aged 12 to 19 years of age were recruited for the study. Several recruitment approaches were used, including advertisement in schools and community settings and peer-to-peer referral in different communities in the district of Kota Bharu. Participants were selected on the basis that they were apparently healthy, without any clinical signs of bone-related disorders or other health problems that might inhibit physical activity, and if they were not currently taking any medications known to influence bone metabolism. One girl was excluded in the final study, because her body dimensions exceeded the bone scanning area. A total of 236 Malay adolescents, comprising 104 boys and 132 girls were finally included in the study.

### Measurements

#### General characteristics and dietary behaviour assessments

A pre-piloted self-administered questionnaire was used to determine socio-demographic and dietary behaviour such as daily breakfast consumption, snacking practices, eating-out practices, and consumption of carbonated and sweetened beverages. This questionnaire, comprised some structured questions and an unstructured open-ended response, where the participants were asked to provide detailed information about their dietary behaviour such as the frequency and place of consumption of common foods and beverages. Frequency of daily breakfast consumption was assessed using an open-ended questionnaire response format, in which participants were asked to report the actual frequency of their usual breakfast consumption. Data were recorded as a continuous variable in the raw form and were then categorized into two breakfast groups namely, frequent breakfast eaters as ≥5 times/week and infrequent breakfast eaters as less than 5 times a week. In addition, the reasons of not taking breakfast daily were also obtained from those adolescents who did not consume breakfast daily. A new quantitative food frequency questionnaire (FFQ) was developed and validated for the present study [18] and was used to assess daily energy intake and other nutrient profiles. In brief, participants were asked to recall the foods consumed during the past year and they then recorded their usual frequency of intake as well as portion size usually served for each food item listed in the FFQ.

#### Body composition profile measurements

Anthropometric measurements such as body weight, height, waist and hip circumferences were assessed according to the standard procedures of the World Health Organization [19]. Each participant was asked to wear light clothing and no shoes during the measurements. Body weight and height were measured to the nearest 0.1 kg and 0.1 cm, respectively, using an electronic scale with attached stadiometer (SECA 220, Germany). Waist and hip circumferences were measured to the nearest 0.1 cm with a measuring tape. Waist circumference was measured at the narrowest point between the lower costal border and the iliac crest at the end of normal expiration, while hip circumference was measured at the maximum circumference of the buttocks in a horizontal plane. All the measurements were taken twice. However if the measurements differed by more than 1.0 cm and 1.0 kg, respectively, a third measurement was taken. The measurements recorded for each participant were the mean values of the two closest measurements.

Body adiposity was assessed using a dual-energy X-ray absorptiometry (DXA) device (GE Lunar Prodigy, USA) at the Department of Medical Radiology at the Hospital Universiti Sains Malaysia. All body scans were performed by one of two trained radiology technicians throughout the study. Quality control for the body scan was performed on a daily basis. The participants were required to wear specific clothing for the DXA scan and removed all metal objects prior to the body scan. Measurements were taken with the participants positioned supine on the scanning table and they were instructed to stay motionless while the ram of the DXA machine passed over the body beginning at the top of the head moving down to the feet. DXA-derived body adiposity levels were expressed as total fat mass in kilograms (kg) and percent of body mass as adipose tissue.

#### Assessment of pubertal Tanner stage

Pubertal growth status was determined by self-reported assessment of breast and pubic hair development for girls and genital hair development for boys according to Tanner pubertal stage classifications [20]. The participant was requested to select the stage that most accurately reflected their current appearance, based on the questionnaire containing illustrations and written description of 5 different Tanner pubertal stages. A random subsample of 20% participants (40 male and 40 female participants) was further examined by the trained personnel of same gender to determine the validity of the self-reported pubertal Tanner assessment tool among these participants. These results showed a high correlation between self-reports of pubertal Tanner assessment and direct physical examination by trained personnel (*r* = 0.971; *P*<0.001), indicating that the self-reported pubertal Tanner stages tool used is able to provide accurate and reliable information on the current sexual maturity of the participants.

#### Assessments of other lifestyle practices

A validated computer-based physical activity (PA) questionnaire to assess the total physical activity levels for the past year was completed by the participants [21]. This questionnaire covers three different physical activity domain namely, activity spent during school time, leisure time and household-based activity. Detailed information on frequency, duration and intensity of each activity for the past year was collected.

### Statistical analysis

Body mass index (BMI) was calculated as weight (kg) divided by height (m) squared and converted to age- and sex-specific BMI z-scores using the LMS method with the UK 1990 growth reference data [22]. Classification of BMI was based on the new revised WHO reference chart for BMI-for-age [23]. All the variables were tested for normality by the Kolmogorov-Smirnov test and test of homogeneity of variance before any statistical comparisons were made. Descriptive statistics were reported as mean values±SD for numerical variables and frequency and percentage for categorical variables, unless otherwise indicated. An independent *t*-test was used to assess the differences between sexes for continuous variables, whereas the Chi-square tests were used for categorical variables. A gender-specific analysis of covariance (ANCOVA) was used to determine the differences in body composition and total adiposity levels according to two breakfast groups, after taking into account other known potential confounding factors such as age, household income, Tanner pubertal status, daily energy intakes, eating-out and snacking frequency and total physical activity levels. Gender-specific analysis was done in order to see any differences of breakfast frequency on body composition between boys and girls. Data analyses were performed using the SPSS for Windows version 18.0 (SPSS Inc. Chicago, IL). A *P* value of less than 0.05 was considered to be significant.

## Results

### General characteristics of study participants

Table 1 shows the general characteristics and breakfast consumption status of the participants. Mean age of the participants was 15.3±1.9 years. The majority (71.2%) were within the normal range for body mass index (BMI). In general, boys tended to have significantly higher levels of body weight, height, and waist circumference compared to their female counterparts. In contrast, adolescent girls had significantly higher DXA-derived body adiposity indices, expressed as total body fat and percent body fat (%BF). Daily energy intake was significantly higher among boys compared to girls with mean intakes of 2346±468 kcal and 2152±547 kcal, respectively (*p*<0.01). The majority of the adolescents reported that they ate food outside of home less than four times a week, while mean snacking frequency was about 2.1±1.1 times a day. Meanwhile, they spent about 1.7±1.4 hours per day in physical activity. A significant difference between boys and girls was in snacking frequency (*p*<0.001) and physical activity (*p*<0.001) with girls reporting higher snacking frequency compared to boys. In contrast, adolescent boys had significantly higher daily physical activity levels.

**Table 1:** General characteristics and body composition profiles of school-aged adolescent boys and girls.

	Boys	Girls	Total
	(*n* = 104)	(*n* = 132)	(*n* = 236)
	Mean±SD		
Age (years)	15.4±1.9	15.2±1.9	15.3±1.9
Household income (RM)	2692±3148	1797±1883^b^	2191±2553
Pubertal growth status% (N)			
- Pre-pubertal (Tanner 1)	5.8 (6)	0.8 (1)^b^	3.0 (7)
- Pubertal (Tanner 2–4)	78.8 (82)	(89)	72.5 (171)
- Post-pubertal (Tanner 5)	15.4 (16)	31.8 (42)	24.6 (58)
Daily energy intakes (kcal)	2346±468	2152±547^b^	2238±522
Eating out practices% (N)			
- Daily	7.7 (8)	5.3 (7)	6.4 (15)
- 4–6 times/week	21.2 (22)	24.2 (32)	(54)
- 1–3 times/week	71.2 (74)	70.5 (93)	70.8 (167)
Snacking frequency (times/day)	1.8±1.0	2.4±1.1^c^	2.1±1.1
Physical activity (hours/day)	2.1±1.7	1.3±0.9^c^	1.7±1.4
***Body composition profile***			
Body weight (kg)	52.5±14.1	48.6±13.4^a^	50.3±13.8
Height (m)	1.6±0.1	1.5±0.1^c^	1.6±0.1
BMI (kg/m^2^)	20.4±4.3	20.6±4.8	20.5±4.6
BMI classification% (N)			
- Underweight	9.6 (10)	10.6 (14)	10.2 (24)
- Normal weight	70.2 (73)	72.0 (95)	71. 2 (168)
- Overweight	20.2 (21)	17.4 (23)	18.6 (44)
Waist circumference	68.0±11.3	65.1±10.3^a^	66.4±10.8
WHR	0.8±0.1	0.7±0.1^c^	0.8±0.1
TFM (kg)	9.9±8.7	16.3±8.9^c^	13.5±9.4
Percentage of BF (%)	17.1±10.0	31.7±8.4^c^	25.3±11.6

BMI = body mass index, WHR = waist hip ratio, TFM = total fat mass, BF = body fat, RM = ringgit Malaysia.

Classification of the BMI was based on the new revised WHO reference chart for BMI-for-age [25].

Significant difference from boys at ^a^ *p*<0.05, ^b^ *p*<0.01 and ^c^ *p*<0.001.

### Breakfast consumption status

Table 2 shows the general breakfast consumption status of participants. About half of the participants (56.4%) reported that they consumed breakfast at least 5 times per week. There was no significant difference found between breakfast consumption frequency by gender (P = 0.154). The majority of these participants took their breakfast at home (72%) while about a quarter of them tended to have breakfast at school (21.6%), with girls more likely to have breakfast at home while boys more likely to have breakfast at a school canteen or stall.

**Table 2:** Breakfast consumption of male and female adolescents.

	Boys	Girls	Total
	(*n* = 104)	(*n* = 132)	(*n* = 236)
	% (N)		
Frequency of breakfast consumption per week			
- ≥5 times a week	61.5 (64)	(69)	56.4 (133)
- <5 times a week	38.5 (40)	47.7 (63)	43.6 (103)
Common breakfast foods^1^			
- bread	63.5 (66)	78.8 (104)	72.0 (170)
- rice dishes	(75)	65.2 (86)	68.2 (161)
- noodle dishes	25.0 (26)	36.4 (48)	31.4 (74)
- sweet and fried traditional cakes	27.9 (29)	27.3 (36)	27.5 (65)
- biscuits	7.7 (8)	21.2 (28)	15.3 (36)
Common breakfast beverages^1^			
- chocolate malt drinks	72.1 (75)	72.7 (96)	72.5 (171)
- tea	57.7 (60)	60.6 (80)	59.3 (140)
- coffee	14.4 (15)	18.2 (24)	16.5 (39)
- milk	9.6 (10)	15.9 (21)	13.1 (31)
- fruit juices	10.6 (11)	7.6 (10)	8.9 (21)
Place of breakfast^a^			
- home	56.7 (59)	83.3 (110)	71.6 (169)
- school cafeteria	28.8 (30)	15.9 (21)	21.6 (51)
- other places (food stalls)	8.7 (9)	0.0 (0)	3.8 (9)

1 Participants may report more than one type of food.

### Relationships between breakfast consumption and body composition profiles

A gender-specific multivariate analysis was used to determine the relationships between breakfast consumption practices and body composition profiles (Table 3). In both genders, frequent breakfast eaters had significantly lower levels of body weight (*P*<0.05), BMI (*P*<0.05), BMI z-score (*P*<0.05), waist circumference (WC) (*P*<0.01), DXA-derived body adiposity measures for total body fat (boys *P*<0.01, girls *P*<0.05) and%BF (boys *P*<0.01, girls *P*<0.05) compared to those of infrequent breakfast practice. These influences persisted even after adjusting for other potential confounders such as socio-demographic status, pubertal growth status, dietary and lifestyle physical activity factors.

**Table 3:** Gender-specific multivariate analysis of the relationships between breakfast consumption status and body composition profiles in male and female adolescents^1^.

	≥5 times a week	<5 times a week	*P*-trend
	Mean±SE		
**Boys**			
n	64	40	
Body weight (kg)	50.0±1.5	56.4±1.9	0.010
Height (m)	1.59±0.01	1.59±0.01	0.990
BMI (kg/m^2^)	19.5±0.5	21.8±0.6	0.004
BMI z-score	−0.19±0.15	0.40±0.19	0.021
WC (cm)	66.3±1.3	70.6±1.6	0.006
WHR	0.79±0.01	0.80±0.01	0.256
TBF (kg)	7.8±1.0	13.1±1.3	0.002
BF (%)	15.1±1.2	20.4±1.5	0.006
**Girls**			
n	69	63	
Body weight (kg)	46.3±1.5	51.0±1.6	0.039
Height (m)	1.52±0.01	1.54±0.01	0.261
BMI (kg/m^2^)	19.8±0.6	21.5±0.6	0.032
BMI z-score	−0.39±0.17	0.21±0.18	0.017
WC (cm)	62.8±1.2	67.6±1.3	0.008
WHR	0.73±0.01	0.75±0.01	0.155
TBF (kg)	14.9±1.0	17.9±1.1	0.048
BF (%)	30.4±0.9	33.0±1.0	0.045

1 adjusted for age (years), household income (RM), pubertal growth status, eating out status (times/week), snacking practices (times/day), daily energy intakes and daily physical activity levels (hours/day).

## Discussion

The main findings of this study indicated that breakfast skipping or infrequent breakfast consumption amongst adolescents was significantly associated with higher DXA-determined total body adiposity, abdominal obesity, assessed by WC and body weight, compared to those who habitually ate breakfast, after taking into account other potential confounding factors. This finding is in line with studies in children and adolescents in Hong Kong and in the United States [16],[24]. An analysis of cross-sectional data in 693 Minnesota adolescents at the end of a 2-year follow-up study showed that adolescents who consumed breakfast more frequently tended to have lower BMI and%BF than those consuming breakfast less frequently [24]. In addition, a recent cross-sectional study in Hong Kong also found that Chinese children and adolescents aged from 9- to 18 years, who were breakfast skippers or only ate breakfast twice or less in a week had higher BMI levels compared to those who were non-breakfast skippers [16]. Furthermore, infrequent breakfast consumption during childhood is associated with higher obesity risk in adulthood as shown in previous studies [25], [26]. The National Longitudinal Study of Adolescent Health carried-out by Niemeier and his co-workers showed that adolescents who skipped breakfast each day during adolescence had a significantly greater risk of developing obesity during the transition into adulthood [25]. Similarly, a recent longitudinal study of children aged 9 to 15 years at the start of the study, when followed over 24 years also found that breakfast skipping in both childhood and current adulthood life were significantly associated with higher levels of BMI, waist circumference and blood markers of insulin and low-density lipoprotein-cholesterol compared to those who were taking breakfast at both time points, suggesting that infrequent breakfast consumption over a long period may have detrimental effects on body weight and cardio-metabolic health [26].

In contrast, several studies with Australian, Portuguese and Saudi Arabian children and adolescents found that there was no significant association between daily breakfast eaters and body weight levels in male and female adolescents [11], [12], [13]. These discrepancies may be partly due to differences in definitions of breakfast skipping and confounding variables used in the final analysis model [27]. However, in the present study, a significant inverse association was found between infrequent breakfast eaters and body composition and body adiposity and this remained statistically significant, even after adjustment for potential dietary and lifestyle confounding factors. The lack of a universal definition for breakfast skipping practices and assessment of the breakfast meal may further complicate the interpretation of the influence of breakfast consumption on health-related outcomes. The definition of breakfast skipping may have a direct impact on the responses given by the participants. For instance, in the previous study, breakfast skipping status was defined as ‘seldom' or ‘never' eating breakfast in a week [11], whereas in the present study it was defined as taking breakfast less than 5 times a week. The use of quantitative classifications, as in the present study, seems to be more helpful in determining the association between breakfast consumption and body composition because self- perception assessment of patterns of eating breakfast in qualitative terms may not be as accurate in reporting actual meal consumption frequency.

Although the mechanisms that explain the influence of infrequent breakfast consumption on body composition are still unknown, there are several plausible theories that could explain such phenomena in adolescents. The habitual skipping of breakfast was associated with poor food choices and unfavorable nutrient intakes [6], [7], [14]. Several studies have shown that children who frequently consumed breakfast tended to consume more fruits and vegetables [6], [7]. Furthermore, breakfast skippers tend to have diets that are high in energy-dense foods and have an increased tendency of overeating at other meals during the day [7], [28], [29]. Several studies have also reported that breakfast skipping was closely associated with dieting practices in girls, because of concerns about body weight and dissatisfaction with their body shape [30], [31], [32]. In the present study, almost half of the adolescent girls reported skipping breakfast because of concerns about becoming fat (data not shown). A similar observation was reported in school-aged adolescent girls in the United Kingdom [8]. It was generally found that adolescents, especially girls, believed that skipping breakfast was an effective method of dieting to lose weight and reduced daily energy intakes [33]. However, our findings show that infrequent breakfast eating was significantly associated with higher body adiposity levels compared regular breakfast consumption.

As the trend towards breakfast skipping has been increasing in children and adolescents, the detrimental effects of breakfast skipping on their health have become increasingly recognised [32]. Therefore, more effort should be made to encourage healthy daily breakfast consumption practices in growing children and adolescents, when behavioural patterns are becoming established during this critical stage of life. One approach could be to introduce healthy breakfast intervention programs at school, as most growing children and adolescents spend much of their time at school. Therefore, active involvement of school authorities, food providers and health personnel would be important to ensure that a successful school breakfast intervention program could be implemented. Continuous monitoring and evaluation would also be needed to ensure that such a program was successful. Healthy eating campaigns at school would not only benefit the students, but may also help their parents in choosing healthy dietary habits at home. Nutrition education by teachers is important for encouraging regular and healthful food choices during breakfast among schoolchildren. Thus, more effort should be directed towards increasing the opportunities for children and adolescents to consume a nutritious breakfast by providing a greater variety of healthy foods for breakfast at school. The government should consider subsidizing the provision of healthy breakfast meals for students from socioeconomically disadvantaged groups.

Some limitations of the present study need to be acknowledged. Due to the cross-sectional design of this study, causality for the positive association between infrequent breakfast consumption and body adiposity and abdominal obesity in adolescents cannot be established. Secondly, for this particular study, we did not look at the nutritional quality of the breakfasts consumed. For instance, the nutrients from breakfast consumption could not be calculated as portion sizes of breakfast foods were not recorded. This finding in apparently healthy adolescents should serve as the basis for future longitudinal studies with large populations to investigate the interaction between infrequent breakfast consumption and obesity and risks of metabolic disorders in growing children. However, a particular strength of this study was to determine body composition and body adiposity accurately by dual energy X-ray absorptiometry (DXA), which is now regarded as the reference method to assess the total body soft tissue composition in children and adolescents. It exposes subjects to only low level of ionizing radiation, is easy to operate and is less expensive compared to CT and MRI procedures [34]. In addition, the inclusion of important biological, dietary and lifestyle confounders such as pubertal growth status, energy intake, snacking and total PA levels in the multivariate analysis model are strengths of this study. Lastly, it is important to note that there is little published information on the breakfast habits of Malaysian adolescents. The large sample size with a power of >70% for detecting differences in body composition status between the habitual and infrequent breakfast eaters over a wide age range, therefore gives confidence for the conclusion that breakfast skipping in this population is a significant influence on health.

## Conclusions

The main findings of this study show that adolescents who ate breakfast infrequently had significantly higher levels of total adiposity and abdominal obesity than adolescents who consumed breakfast regularly each day. This indicated that breakfast skipping may increase the risk of excessive body adiposity and as a result, may contribute to the risk of obesity and related metabolic consequences. Therefore, daily breakfast consumption with healthy food choices should be encouraged among children and adolescents in order to prevent excessive body weight gain during their critical years of growth. However, longitudinal studies with large sample sizes would be needed to determine the precise mechanism linking daily breakfast consumption with body adiposity, and health and metabolic-related outcomes in growing children and adolescents.
